# Veratrum Viride

**Published:** 1891-02-14

**Authors:** 


### THERAPEUTICS.

### VERATRUM VIRIDE.

The green hellebore is a plant whose medicinal properties have been recognised in America for many years, but English experience of the drug is decidedly limited. The investigations of Dr. H. C. Wood show that its chief action is on the heart, both by direct action on the heart-muscle and by stimulation of the cardiac inhibitory centres. It is a powerful vascular depressant, decreasing the force of the cardiac contractions, slowing the pulse, and lowering arterial tension. It is chiefly owing to this depressing action on the heart that many practitioners have disliked using it in practice, but when given in small doses dangerous symptoms need not be feared; it must, however, be administered with the greatest caution to patients with broken down constitutions, such as drunkards. It is in pneumonia that the toxic or cardiac depressing action has proved of greatest service. Dr. Wood reminds us that in the early stages the capillaries of the affected portion of the lung are dilated and gorged with blood, the rapidly acting heart with powerful ventricular contractions tending to increase the local inflammatory processes. The action of veratrum is to dilate the arteries generally, and perhaps especially the capacious abdominal vessels, as well as to markedly diminish the heart's action, and thus the blood is drawn into the abdominal vessels. In other words, it bleeds the patient into his own vessels, and we get by its administration all the advantages, and none of the drawbacks, of the old methods of vivisection. Veratrum should be given in the early stages only of pneumonia; when consolidation and exudation have taken place it is too late, and, moreover, the patient cannot then be safely brought under the influence of a cardiac depressant. Veratrum has also been advocated in other inflammatory affections, especially in gout, acute rheumatism, and similar diseases. It has been used in acute tonsilitis, for which Dr. Hudson is most emphatic in his praise of the drug, especially in combination with morphine. He finds that the liability to nausea produced by either drug is greatly diminished by administering them together. Thus he sees reason to believe that they exert therapeutic properties when combined that are not observable when either is administered alone. The following mixture is recommended: ℞ Tincture of veratrum viride ♏ xxx.; sulphate of morphine, gr. is.; distilled water, ℥iv. A teaspoonful to be given twice, with an interval of an hour: then every two or three hours as required. But veratrum viride, like all drugs which, when administered in large doses, are cardiac depressants, has a primary stimulating action on the heart. This action has been employed with most gratifying results by Christovitch. He used a one per cent. solution, the dose being from ten to twenty drops. He found that the drug increases in cardiac contractions, rendering them more forcible, and that the pulse becomes fuller. The secretion of the urine is increased in amount, and dropsical exudations in various parts of the body disappeared. Congestions of the lungs, liver, and kidneys were either cured or relieved by the remedy. In those cases in which there was great hypertrophy of the heart, associated with violent palpitation which is purely functional, such small doses decrease the heart's action. It will also relieve the palpitation due to irritation of the pneumogastric in gastric catarrh, but is inferior in this respect to some other remedies.

